# A Practical Essay on Diseases and Injuries of the Bladder

**Published:** 1823-03-01

**Authors:** 


*A Practical Essay on Diseases and Injuries of the Bladder, being that to which the Royal College of Surgeons adjudged the Jacksonian Prize for the Year* 1821*: Irritable Bladder is treated of in all its Varieties, both with and without Mucous Discharges; also, Inflammation, Suppuration, and Ulceration of that Organ; with Cancer and Stone, the Formation of which last is explained on entirely New Principles: Retention and Incontinence of Urine are considered very fully; and the whole is prefaced by an Inquiry into the Mutual Influence that exists between Life and Organization; including some Observations on Ocular Spectra and the Nature of Mind.* By Robert Bingham, Fellow of the Royal College of Surgeons; Author of Practical Essays on Strictures of the Urethra and Diseases of the Testes, &c. &c. and Lecturer on the Theory and Practice of Surgery. Octavo, pp. 467. London, October, 1822.

This Essay was composed in a great hurry, the author having only three months to prepare it for adjudication at the College. Its bearing off the prize, is a proof that it was the best essay offered at that time, and such sanction is, undoubtedly, a better reason for coming before the public at large, than most authors can offer.

Mr. Bingham thinks, that a digest of what has hitherto been advanced upon the subject of vesical diseases, scattered as the materials must be throughout the writings of so many authors, has long been a desideratum in surgery. Independently, however, of bringing the rays of knowledge into a focus, our author hesitates not to assert, "that this volume contains much new matter, with respect both to the nature and treatment of the diseases in question." A few of these novelties are glanced at in the preface. One is, the shewing of disease in the urinary bladder, originating in consequence of ulceration in the large intestines. Another claim to originality is, the pointing out of spinal affections as productive of irritability and mucous secretion in the bladder. On retention of urine, he asserts that, much new and important information is communicated. "Hitherto it has never been explained, or even suspected, that retention of urine sometimes proves fatal in consequence of a vicarious secretion taking place into the lungs, and causing suffocation." When we come to the subjects individually, we shall probably shew, that Mr. Bingham is not quite so original as he supposes himself to be, on some of the points abovementioned.

The work is prefaced by "an Inquiry into the Influence that exists between Life and Organization." We certainly can see little occasion for this metaphysico-physiological proem to a work on the bladder; and think Mr. B. would be wise to cut it out of his next edition. He states that, in the preface to his work on Strictures, he has *proved* organization "to be the effect of *pre-existent* life acting on unorganized matter." Now, although we hope and believe, that mind may exist independent of matter, yet, as we never did, and never can, see mind or life unconnected with organization, there cannot possibly be any *proof* shewn of their separate existence. It can only be a matter of belief, or a matter of speculation. So far Mr. Bingham's confident expressions about *proofs*, are not well calculated to prepossess us much in favour of his reasonings on this, the threshold of the work.

A considerable portion of this inquiry is taken up in the description of a kind of natural kaleidoscope or phantasmagoria, which our author has discovered by lying on his back, closing his eyes, and pressing firmly upon the centre of each with the point of his finger. By this operation, he produced a succession of ocular spectra, more numerous than "autumnal leaves in Vallambrosa," and of which he has laboured to give a description, but evidently in vain.

"The deep hue and brilliancy of the colours and the general sparkling, beautiful appearance of the whole spectrum baffles description. I can think of nothing which bears any comparison with it unless it be a piece of Mosaic work, composed of the finest gems, rubies, emeralds, diamonds, &c."—*Inquiry*, xlv.

We are really surprized, that a man of Mr. Bingham's sense, could bring himself to fill so many pages with descriptions of such ridiculous phantoms; and, as for the theories growing out of them, they appear to us to be, in every sense of the word,—completely *visionary*. It is a pity our author did not transmit his new phantasmagoria to the College with the prize essay, to be deposited in the museum for the amusement of certain old "fellows" who are fond of optical illusions.

But we must leave the region of fancy, and descend to the region of facts. Under about seventy heads, Mr. Bingham descants on as many diseases or operations appertaining to the urinary bladder. Another volume is to follow on the prostate gland, and our readers are aware, that a volume on the urethra has preceded. England is now the land of monographs; and the ancient rage of system-makers is almost extinct.

*Irritable Bladder.* This complaint is very often sympathetic of diseases not in the bladder, but in the urethra, prostate, uterus, vagina, rectum, kidneys, and in the digestive organs. In such cases it will be needless to aim at relieving the symptom without removing the cause. Several cases are related by Mr. Bingham of irritable bladder from derangement of the alimentary canal, and which were cured by regulating the diet, correcting the secretions by alteratives, and giving tone to the digestive functions by vegetable bitters. The cases of supposed ulceration in the colon producing irritable bladder, appear to us doubtful. We can hardly think that the disease amounted to actual ulceration. Patches of a purulent-looking matter on the fæces are no proofs, to our certain knowledge, of ulceration in the gut. Speaking of that irritability of bladder which not unfrequently attends severe gonorrhœa, our author states that he has invariably found it to be relieved by light tonic medicines. Our author introduces cases of irritable bladder from diseases in the various neighbouring organs abovementioned, and then proceeds to—

*Irritable Bladder with Mucous Discharge.* Irritability without discharge shews that only the nervous system of the bladder is affected—when a secretion of mucus takes place from the internal surface, it is a proof that there is an increased vascular action. Mucous secretion from the bladder is met with in four different states—like a jelly adhering all round the inside of the pot de chambre—puriloid, so as to be with difficulty discriminated from pus—glairy and exceedingly tenacious—and mixed with an earthy matter which has been compared to hair-powder.

To determine whether there be a morbid secretion of mucus from the bladder, the urine should be examined immediately after it is voided; for when cold it often throws down a sediment which may be mistaken for mucus. If only cold urine can be procured it should be heated to a vital temperature; and if mucus still appear, it will be presumptive proof, though its disappearance by heat is no certain proof to the contrary. We do not think it possible in all cases to distinguish pus from puriloid mucus; nor is it of much consequence, since we know that real pus may be secreted from an unbroken surface.

*Etiology.* There can be no doubt entertained in the present day, that the immediate cause of catarrhus vesicæ (the name given by Lieutaud, and perhaps the best) is irritation or inflammation of the mucous membrane of the bladder. The causes that determine this state of the membranes are numerous, and may be divided into general or sympathetic, and local. Mr. Bingham observes that the application of cold to the surface of the body must be a very rare cause of this complaint. We do not think so. Nothing is more usual in cases of common colds, than a temporary mucous discharge in the urine—an observation which every old woman in the kingdom is in the habit of making. Our soldiers, when exposed to great cold and wet at nights, by bivouacing in the open air, are not uncommonly affected for a time with catarrhus vesicæ. Immoderate exercise, or the contrary, a sedentary life, great mental exertion, the depressing passions, and old age itself are all occasional causes of the disease. Derangement of function in the digestive organs, if long continued, may produce this sympathetic affection of the bladder as well as irritability. The use of very stimulating diuretics, as the lytta, the suppression of periodical discharges, as of perspiration of the feet or the hæmorrhoidal flux, has been known to produce catarrhus vesicæ, and so has the imprudent repulsion of cutaneous eruptions. Metastases of rheumatism and gout may be reckoned among the causes of this complaint.

Among the local causes we would place in the first rank, obstructions to the free discharge of urine along the urethra, and irritation propagated from that passage to the bladder. We imagine that the rarity of the disease in former times, and its frequency in our days, may be accounted for by the prevalence of gonorrhœal affections within the last three or four hundred years.

"My own experience," says Mr. Bingham, "would lead me to think that it (catarrhus vesicæ) results most certainly from the irritation of some portion of urine being always retained in the bladder, and this seems to be the case whether the retention be caused by paralysis of the bladder, or enlargement of the prostate gland."

Now we really cannot see the force or the ingenuity of this doctrine. If indeed the urine were retained so as to make painful distention of the bladder, and thereby keep up irritation, we would set it down as a cause; but the mere retention of a small residue at each evacuation, does not appear to us to be an adequate cause. The inner coat of the bladder is always in contact with urine. The receptacle is no sooner emptied than new urine begins to trickle down from the kidneys, so that the mere presence of urine cannot be irritating. It is the *distention* of the bladder, or the *force* which it may be necessary for it to exert in discharging the urine that appears to us to be the main cause in these instances.

Our author's Essay on Catarrhus Vesicæ is very incomplete. He gives little or no symptomatology or history of the complaint—no division of the disease into acute and chronic—and no *post mortem* appearances. These are striking defects. The *acute* form of the disease is generally preceded or accompanied by a febrile movement in the system, and by some heat, pain, or sense of tension in the hypogastric region. To these succeed frequent inclination to make water, and some difficulty in making it. The urine itself, on standing, deposits a mucous sediment. These symptoms continuing, with more or less intensity, for some days, either gradually subside; or the febrile and inflammatory phenomena disappearing, the local ones continue, viz. the irritability of the bladder, and the secretion of mucus. Chronic catarrhus vesicæ is then established.

This last form of the disease, however, which is far more common than the acute, generally creeps on in so slow and insensible a manner that no febrile or inflammatory symptoms are recognized. The causes may nevertheless be the same as those of the acute species. Calculous concretions of an angular or irritating figure, are not unfrequently the causes of chronic cat. vesicæ, which is more commonly found among elderly people, and those who had been afflicted with gout, rheumatism, or cutaneous complaints. The quantity of mucous sediment in this species varies much, being sometimes a quarter, a third, or even half of the whole fluid discharged from the bladder. When this mucous discharge is considerable, it is generally accompanied by emaciation, but seldom by fever or constant pain in the urinary apparatus. The principal inconveniences which it produces are the frequent inclination to make water, and sometimes the difficulty of discharging it, when it is very ropy. In this state, and when not occasioned by a foreign body in the bladder, the disease may continue for years, and even for the greater part of a person's life, without proving fatal. At the same time, although there are instances of a cure being effected in this complaint, it must be confessed that such a fortunate issue is rarely to be expected, especially in elderly people.

*Dissection* presents us with a quantity of fetid and mucopurulent urine in the bladder, the tunics of the organ being thickened, and its mucous lining studded with numerous glandular bodies, from which the same kind of glairy mucous fluid which passed with the urine, can be squeezed. Ulceration of the interior coat is also occasionally found, and enlargement or other disease of the prostate gland.

In the treatment of vesical catarrh, whether acute or chronic, it is necessary to make a diligent scrutiny into the cause of the complaint; for by directing our agents towards the original source we have a better chance of success. But whatever the cause, one important indication is to allay the irritation and inflammatory state of the bladder itself. Where the inflammatory symptoms are pretty well marked, blood should be taken from the loins by cupping, or leeches applied to the pubic region, followed by emollient fomentations and hip-baths. The drink should be of the most unirritating and diluting kind, as linseed or marshmallow tea, barley water with gum, &c. If any difficulty in the passage of the urine occurs, the catheter should be introduced; for nothing is so injurious to an inflamed bladder as straining to evacuate its contents. Anodynes may also be employed, where the irritation is considerable, especially the hyoscyamus, conium, and lactucarium. The digestive organs must be strictly attended to. Food must be of the blandest and least stimulating kind, and daily evacuations procured by the most simple aperients, as castor oil or the neutral salts.

Some authors, and especially Foote and Wadd, have recommended injections into the bladder, and we think this remedy is too much overlooked. Chopart injected the liquor plumbi acetatus dilutus into the bladder of an old man, 75 years of age, who was exhausted by an excessive catarrhus vesicæ. The glairy discharge was greatly restrained, the patient acquired strength, and lived two years afterwards.

For the cases of catarrhus vesicæ related by Mr. Bingham we must refer to the volume itself.

*Inflammation of the Bladder.* Mr. Bingham's description of this complaint is very imperfect. He does not divide it into the acute and chronic forms—at least, he only quotes *Hoffman's Practice of Physic* for cases that existed several years. The following is our author's symptomatology of cystitis.

> "This is denoted by tumor, and burning pain in the hypogastric region; tenderness both above the pubes and in the perineum; incessant desire to make water, with violent straining; vomiting, tenesmus, and frequent pulse; great restlessness, wild expression of the eyes, and occasional delirium, and sometimes retention of urine." P. 91.

We should not be inclined to place the hypogastric *tumour* in the front rank of symptoms, as it occurs only in consequence of the retention of urine, which last symptom is itself only a consequence of inflammation about the neck of the bladder. The same etiology which was described in catarrhus vesicæ is applicable to cystitis, which is only an extension of the inflammation from the internal to the whole of the coats of this viscus. The treatment is the same as for the acute catarrhus vesicæ, only that it is to be more energetic in degree.

Chronic inflammation of the bladder is sometimes a sequel of the acute form, and sometimes steals on, like chronic vesical catarrh, by imperceptible degrees, at the beginning. Its symptoms do not materially differ from those of the last mentioned disease, and it generally has a fatal termination, by ending in complete disorganization of the bladder. The following are the usual appearances on dissection. The capacity of the organ is greatly diminished, and in its contracted cavity is found a mixture of urine and puriform, or muco-purulent matters. The coats of the bladder are greatly thickened, and indurated sometimes to an almost horney consistence. The coats are often found an inch or even two inches in thickness, and the muscular tunic developed in an extraordinary degree, the cavity sometimes not larger than would contain a crab-apple. On the other hand, the volume of the bladder has been found enlarged to the size of a child's head, by the thickening of the parietes, the internal cavity not being larger than that of an egg. When the inflammation has occupied much of the peritoneal covering of the bladder, extensive adhesions are found to exist between it and the neighbouring parts. Excepting where the cause is a calculus, and the operation is performed, the treatment can only be palliative, and may be easily gathered from what we have said of chronic catarrhus vesicæ.

*Retention of Urine.* "Some authors," says Mr. Bingham, "have written that retention of urine is a disease of great urgency and danger, but this is not correct: for, strictly speaking, it is no disease at all. It often is the effect of disease, and it often is a cause of disease; but, as before stated, it does not of itself constitute a disease." We really do not comprehend this mystery, and Mr. Bingham has not condescended to explain it. In the very same page he virtually contradicts the above statement, representing retention of urine as sometimes coming on suddenly, "under which circumstances it is generally most complete and *most alarming;* for, unless it be speedily remedied, it runs its course, and *terminates the life of the patient in a few hours, &c.*" How does this tally with the first sentence of the section that it is not a disease of great urgency and danger—in fact, that it is no disease at all! We can readily allow that retention of urine may be the effect of inflammation or stricture; but surely Mr. Bingham will not persuade us that, when the retention takes place, it is not a serious disease in itself, and equal to the destruction of life, whereas no such event would take place, were it not for the supervention of this phenomenon.

The subject of retention, and the operations necessary for it, especially catheterism, occupy nearly a fourth of the volume, and from the want of order, the multiplicity of divisions, and the introduction of tedious cases, are spread out to a most tiresome length, and yet not containing full information on the point under discussion. The following is our author's etiology of the complaint.

"*Causes.* These are of two kinds, either the want of expulsive power in the bladder, or some insurmountable obstruction that prevents the flow of the urine. The want of expulsive power may be either a total paralysis or mere loss of tone, in consequence of the detrusor muscles having been sprained. The mechanical obstructions may be numerous, such as spasms and inflammation of the sphincter vesicæ, or of the membranous part of the urethra; a coagulum of blood or stone in the bladder; a calculus lodged in some part of the urethra; strictures of that canal; diseases of the prostate gland, or some imposthume forming near the anus." 129.

As it is of great importance to be well acquainted with the various causes of urinary retention, we shall enlarge a little on this subject, and cull information from other sources than the work under review.

Mr. Bingham takes no notice, that we see, of renal retention of urine, a complaint by no means uncommon. The ureter is liable to inflammation like other membranous canals, and to have its calibre lessened or obstructed by thickening of its coats, by coagula of blood, by calculi, or by tumours in the neighbourhood. In such cases the ureter, above the obstruction, dilates, and ultimately the kidney itself; some remarkable instances of which we lately gave in this Journal. Callisen and other writers have found several pints of urine in the pelvis of the kidney, and Desgranges reports instances where the ureter was enlarged to the size of the colon, forming convolutions and pouches of great magnitude. Unfortunately the symptoms of this malady are equivocal, and therefore we shall not dwell upon them; but the young practitioner should know that such diseases will occasionally cross his path, and the appearances on dissection should not take him by surprize.

*Vesical retention*, then, is the disease with which we have most to do, and its etiology is very extensive. Calculi in the bladder sometimes cause retention of urine, and more especially small calculi that happen to get jammed in the passage. In such cases the calculi must either be pushed back with the catheter, or extracted by means of the admirable forceps invented by Sir Astley Cooper and Mr. Weiss.

A coagulum of blood in the bladder is not a very rare cause of retention, especially after lithotomy. It requires the injection of warm water through the wound or urethra, and then the urine to be drawn off by a catheter. The same observations will apply where a collection of glairy mucus obstructs the passage and causes retention of urine, in some cases of catarrhus vesicæ.

*Irritation or Inflammation* of the bladder itself, especially of the neck of the bladder, is perhaps one of the most frequent causes of retention of urine, and this inflammation, congestion, or irritation may be determined by all the causes, local and constitutional, which we have alluded to as productive of vesical catarrh. It may be permitted us here to remark, that when this cause of retention is present, it will be prudent to reduce, in a considerable degree at least, the inflammatory condition of the parts before catheterism be employed. This reduction will, of course, be effected by blood-letting, general and local, fomentations, glysters, antimony, and finally, anodynes. The catheter will then be much more easily introduced.

*Paralysis of the Bladder* is not an infrequent cause of retention, especially in elderly people, and it is astonishing to what a size the receptaculum urinæ will sometimes reach, under these circumstances. Instances are related by Lieutaud, Murray, Baldinger, and Beddingfield, where the bladder has contained fifteen, sixteen, and even twenty pints of water. It is well known that in these cases the patient goes on making water, but without ever completely emptying the bladder, and when the viscus arrives at a certain stage of distention, there is a constant dribbling, or an incontinence rather than a retention of urine.

The causes of this complaint are sometimes manifest, sometimes obscure. Over-distention, old-age, affections of the spine, excess of venereal indulgences, onanism, hard labour, are among the principal causes. It is evident that the catheter is our principal, perhaps only resource in such cases. But we would recommend our young surgical brethren not to content themselves, as they sometimes do, with drawing off the urine every twelve hours. They should draw it off every six hours at least; but the best plan is to leave the catheter in the bladder, and thus let the organ keep in a constant state of contraction. Richerand and some other surgical writers have exaggerated the dangers attendant on leaving a catheter in the bladder, by supposing that air will get into that organ, and cause irritation or inflammation. This, we believe, is quite imaginary.*

Of tumours and other disorganizations about the neck of the bladder, determining retention of urine, it is not necessary to speak here.

*Urethral Causes of Retention.* These are the most numerous class perhaps of all. Calculi, strictures, foreign bodies, clots of blood, inflammations, organic changes—all these in turn produce retention of urine, and require modifications of treatment according to the cause.

But we must now give some account of our author's observations on retentions.

He remarks that, if retention of urine be not relieved by art, it generally proves fatal. Most usually the bladder ulcerates, or rather sloughs, and the urine escapes into the neighbouring parts. The retention of urine, however, sometimes proves fatal without any extravasation of urine or breach of continuity in the bladder. Mr. Bingham asserts that the bladder is *never* lacerated by over-distention. This is a hardy assertion. William Hunter relates the case of a poor woman, whose bladder burst and discharged eight or nine pints of water into the abdomen, which quickly proved fatal. M. Deschamps relates a similar case in the 48th volume of the Dict. des Sciences Medicales, page 123. We believe, however, that these instances are very rare, and that the ulcerative process is the usual mode of the bladder's giving way.

The comatose state of brain which usually takes place in fatal retentions of urine, does not, he supposes, depend on *translation of urine to the brain*, but is entirely owing to a determination of blood. As all the excretions, and also the effusions on the brain, in such cases, exhale a urinous odour, we do not see any great improbability that there should be an absorption of urine into the blood, the same as of bile in jaundice, and thus irritation of the brain produced. One of Mr. Bingham's discoveries, as noticed in the preface, is the suffocation of some patients labouring under retention of urine, "in consequence of the air-cells of the lungs becoming filled with frothy fluid." Of the exclusive originality of this termination we doubt, for we see Montfalcon allude to it in the article "retention," in the Dict. des Sciences Medicales, without appearing to consider the observation as any thing new.

* In these paralytic cases much may be done in aid of catheterism by frictions with the lytta; and also by a judicious administration of the tinctura lyttæ internally.

But be that as it may, the case on which the doctrine is founded, does not appear to us to have a single symptom of retention of urine attached to it. Our author was called to a muscular man whom he found stretched out in bed perfectly insensible, with strong indications of congestion in the brain, his pulse 120, hard and full. His wife informed Mr. B. that he had only eaten and slept alternately for several days; and that about twelve hours previously, he had taken for breakfast, half a pound of beef-steaks, with a proportion of ale, and several glasses of gin.

"I enquired how he made water, and his wife replied very well, and assured me that she had seen him void a pint or more of colourless urine at one time that very day." 116.

Mr. B. drew off forty ounces of blood from the temporal artery, which brought him to his senses. Next morning he was found insensible—his breathing making a noise as if the air was forced through mucus, and much frothy fluid issuing from his mouth and nostrils. He died in a few hours. On opening the head, the vessels of the brain were found gorged with blood—the ventricles were deluged with serum—kidneys inflamed—"*the bladder appeared to be perfectly healthy; it contained a little more than a pint of limpid urine, which distended it to feel tense as a drum head.*" Mr. B. found a stricture in the urethra, through which he could only force a very fine stream of urine. Now, notwithstanding the drum-head distension, produced by a pint of limpid urine, and the difficulty which our author found in forcing a stream through a dead urethra, we appeal to our surgical brethren, whether there was the slightest reason to put this case down as one of fatal retention of urine? No examination was made of the lungs, and on this case, is erected the novel theory to which our author draws the attention of the profession in his preface. That the man died of congestion and serous effusion in the brain, we have no doubt;—and, that the urinary organs had any thing to do with the fatal termination, we see no evidence.

In the treatment of retention of urine, our author recommends, under all circumstances, when the cause is inflammation or spasm, the warm bath, mild but active purgatives, anodynes, and, perhaps, bleeding. By "bleeding," he means venesection; for he properly recommends local bleeding in cystitis and in this kind of retention of urine. From having been told by some patients "who had *complaints in the urethra*, that they found much greater relief from bathing with cold water, than from using water that was warm," our author is disposed to think, that some cases of *retention of urine* would be most relieved by cold water. This is loose reasoning in a practical prize essay. Our author, we think, is judicious in pointing out the impropriety of indiscriminate recourse to the catheter as the first measure in every case of retention of urine. "Retention of urine, (says Mr. B.) resulting from inflammation and spasm, may almost always be relieved by means of the warm bath, anodyne enemas, mild but active purgatives, and the abstraction of blood." This was the opinion of Pott and Heister. Mr. Bingham has no opinion of the muriated tincture of iron in retentions of urine; and on enquiry among his most intimate friends, the same opinion was elicited. As we have never trusted to this remedy alone, in such cases, we cannot say what is the exact power which it individually possesses in relaxing spasm about the urinary passages. The infusion of tobacco he prefers to the smoke.

*Catheterism.* Our author makes some judicious remarks, on the process of instrumental aid in retention of urine, but they are excessively diluted, as usual, with words and cases, not always the most illustrative. Where the retention has been caused by a voluntary abstinence from discharging the urine too long continued, Mr. Bingham thinks, that we cannot too soon have recourse to artificial means of emptying the bladder, "for, in these instances, the spasm of the sphincter vesicæ having been first occasioned by powerful volition, is afterwards continued in consequence of irritation from urine." There are some cases, he observes, where a kind of valve is formed over the internal orifice of the urethra, which confines the urine more and more perfectly, in proportion to the violence of the efforts to expel it. These cases can only be relieved by instruments. Elastic gum catheters our author prefers to silver.

"Used on a stilet they may have the same degree of firmness imparted to them as belongs to the silver ones, and owing to their pliability, if employed without a stilet, they may frequently be successfully introduced in the same manner as a bougie, which is by far the simplest operation of the two, and often constitutes the only means by which an unpractised operator will be able to succeed." 158.

Mr. Bingham considers it a good plan, to keep the elastic gum catheter on stilets that are much bent, for, by being retained a long time in this shape, they acquire a degree of curvature of their own, which makes them, he avers, very superior instruments for some patients, especially those affected with prostatic diseases.

Here we must stop; for, on running over the remainder of the volume, we found that we could glean little that was worth extracting. Upon the whole, we have been greatly disappointed in this prize Essay, and we do not apprehend, that Mr. Bingham will gain much, in any way, by its publication. We do not think that any but hospital surgeons, or those in very extensive private practice, should take upon themselves to write on such subjects as those contained in the volume before us, unless as professed compilers. We object, also, to the manner in which our author has stated his cases. They are all, or almost all, unauthenticated by names, dates, or places—an omission which no writer ought to make, unless on some very particular occasions, where the names of the parties could not, with propriety, be revealed. We should suppose, that no such very great delicacy was necessary in the majority of cases introduced in the work under consideration.

